# Multiple Herbicide Resistance in *Lolium multiflorum* and Identification of Conserved Regulatory Elements of Herbicide Resistance Genes

**DOI:** 10.3389/fpls.2016.01160

**Published:** 2016-08-05

**Authors:** Khalid Mahmood, Solvejg K. Mathiassen, Michael Kristensen, Per Kudsk

**Affiliations:** Department of Agroecology, Faculty of Science and Technology, Aarhus UniversitySlagelse, Denmark

**Keywords:** genotyping, herbicide resistance, metabolic based resistance, promoter analysis, regulation of expression, weed management

## Abstract

Herbicide resistance is a ubiquitous challenge to herbicide sustainability and a looming threat to control weeds in crops. Recently four genes were found constituently over-expressed in herbicide resistant individuals of *Lolium rigidum*, a close relative of *Lolium multiflorum*. These include two cytochrome P450s, one nitronate monooxygenase and one glycosyl-transferase. Higher expressions of these four herbicide metabolism related (HMR) genes were also observed after herbicides exposure in the gene expression databases, indicating them as reliable markers. In order to get an overview of herbicidal resistance status of *L. multiflorum* L, 19 field populations were collected. Among these populations, four populations were found to be resistant to acetolactate synthase (ALS) inhibitors while three exhibited resistance to acetyl-CoA carboxylase (ACCase) inhibitors in our initial screening and dose response study. The genotyping showed the presence of mutations Trp-574-Leu and Ile-2041-Asn in ALS and ACCase, respectively, and qPCR experiments revealed the enhanced expression of HMR genes in individuals of certain resistant populations. Moreover, co-expression networks and promoter analyses of HMR genes in *O. sativa* and *A. thaliana* resulted in the identification of a *cis*-regulatory motif and zinc finger transcription factors. The identified transcription factors were highly expressed similar to HMR genes in response to xenobiotics whereas the identified motif is known to play a vital role in coping with environmental stresses and maintaining genome stability. Overall, our findings provide an important step forward toward a better understanding of metabolism-based herbicide resistance that can be utilized to devise novel strategies of weed management.

## Introduction

*Lolium multiflorum* L. (Italian rye grass) originated in the Mediterranean region (Inda et al., 2014), however it is distributed worldwide and poses weed management problems in many different cropping systems including winter cereal crops in Europe (Stanger and Appleby, 1989). It is one of the weed species that are most prone to evolve resistance to herbicides (Heap, 2016). Introduction of acetyl coenzyme-A carboxylase (ACCase) herbicide in the 1980s enabled control of *L. multiflorum* in wheat fields (Stanger and Appleby, 1989). However, the continuous use of ACCase herbicides caused evolution of resistance in *L. multiflorum* populations. Shifting to acetolactate synthase (ALS) inhibitors eventually also led to the evolution of ALS resistant *L. multiflorum* populations. ACCase resistant *L. multiflorum* was reported for the first time in the USA in 1987, whereas resistance to ALS inhibitors was documented in 1995, also in the USA (Heap, 2016). Since then 55 incidences of herbicide resistance in this species were documented in 12 different countries to 7 different mode of actions of herbicides (Heap, 2016). In Denmark, herbicide resistance in *L. multiflorum* was reported in 2010 for the first time (Mathiassen, 2014).

Herbicide resistance is an evolutionary process and a classic example of rapid dynamic adaptation to human mediated selection pressure (Powles and Yu, 2010; Neve et al., 2014). This evolutionary process largely depends on the biology of the weed species, the biochemical properties of herbicides, the number and rate of herbicide application, management factors, and genetics such as the frequency of resistant alleles and their associated fitness cost (Powles and Yu, 2010; Délye et al., 2013; Carroll et al., 2014). Analysis of herbicide resistance through biochemical and genetic means revealed coexistence of one to several resistance mechanisms. These resistance mechanisms can be divided into target-site resistance (TSR) and non-target site resistance (NTSR) mechanisms, (Yuan et al., 2007; Délye, 2013; Délye et al., 2013). TSR occurs by mutation within a single gene coding for a herbicide target-site enzyme or by overproduction of the target enzyme, whereas NTSR involves mechanisms that minimize or prevent the amount of herbicide reaching the target site (Délye, 2013; Délye et al., 2013). TSR is relatively easy to study due to their monogenetic inheritance that are often well-documented in herbicide resistant weeds, whereas this is not the case for NTSR (Devine and Shukla, 2000; Han et al., 2016). NTSR is usually considered to be polygenic inherited due to the involvement of many genes such as cytochrome P450 monooxygenase (P450s), glucosyl transferases (GTs), glutathione *S*-transferases (GSTs), and/or esterases and ABC transporters (Yuan et al., 2007; Yu and Powles, 2014). There is scarcity of information about the exact gene or genes endowing metabolic based herbicide resistance. Recently a few genes have been identified applying next generation sequencing technology (Gaines et al., 2014; Duhoux et al., 2015; Gardin et al., 2015). Gaines et al. (2014) found four consistently over-expressed genes in resistant individuals of *Lolium rigidium*, a close relative of *L. multiflorum*. These include two- cytochrome P450 (P450), one nitronate monooxygenase (NMO) and one glycosyl-transferase (GT). Regulatory proteins known as transcription factors control the expression of these herbicide metabolism related (HMR) genes (Sullivan et al., 2014) but nothing is known about them in the context of metabolic based herbicide resistance. Public transcriptomic databases have valuable information of thousands of independent experiments and can be utilized to predict regulatory network with recent bioinformatics tools. This strategy has been employed to predict essential components of several signaling or metabolic pathways in various organisms ranging from prokaryotes to eukaryotes (Vadigepalli et al., 2003; Li et al., 2006; Hirai et al., 2007; Suzuki et al., 2009; Muraro and Simmons, 2016).

Given the relevance of *L. multiflorum* as a major weed of cereal crops and the significance of herbicide resistance in this weed species, it is important to elucidate the resistance mechanisms prevailing in Danish fields. Hence the first objective of the present study was to find out the resistance mechanisms in *L. multiflorum* populations in general and to identify specific metabolic based resistant populations in particular. The second objective of our study was to identify regulatory proteins responsible for expression of HMR genes through using various bioinformatics tools and analyzing data of independent experiments deposited in global databases. For this purpose, we studied the promoters of genes to link them with probable transcription factors and identification of conserved motif. Furthermore, patterns of expression profiling were investigated to gain deeper insight about role of identified regulatory proteins. To the best of our knowledge, this is the first attempt in order to mining the transcriptional regulation of metabolic based herbicide resistance in any weed species.

## Methods and materials

### Plant material

Seeds of 19 populations of *L. multiflorum* were collected. Among them 18 populations were sampled from different locations of Denmark, whereas one population (ID-27) was obtained from France. The majority of populations were collected from fields where unsatisfactory control of ALS and ACCase inhibitor herbicides had been observed. The *L. multiflorum* is not an endangered species and no specific permit is required for collection of seeds from agricultural fields. Two known susceptible populations ID-290, a population maintained by the Department of Agroecology, Aarhus University and the commercial forage grass variety Sikem were used as standard reference populations.

### Herbicide application

Two herbicides, iodosulfuron-methyl-sodium and clodinafop-propargyl, belonging to the groups of ALS and ACCase inhibitors, respectively, were applied through foliar application to *L. multiflorum* plants at the 3–4 leaves stage. The common trade names of the herbicides are Hussar OD (100 g L^−1^ iodosulfuron-methyl-sodium + 300 g L^−1^ mefenpyr, Bayer Crop Science, Denmark) as an ALS inhibitor and Topik (100 g L^−1^ clodinafop-propargyl + 25 g L^−1^ cloquintocet-mexyl, Syngenta, Denmark) as an ACCase inhibitor. All spray solutions were prepared in deionized water and applied in mixture with 0.5 L ha^−1^ Renol (vegetable oil) using a laboratory pot sprayer fitted with two Hardi ISO F-110-02 nozzles. The nozzles were operated at a pressure of 310 kPa and a speed of 5.5 km ha^−1^ delivering a spray volume of 150 L ha^−1^. Herbicides solutions were prepared at 2 g a.i. ha^−1^ iodosulfuron-methyl sodium (≈0.0013%) and 25 g a.i. ha^−1^ clodinafop-propargyl (≈0.0167%). Plants were harvested 4 weeks after herbicide application and fresh weights were recorded.

### Herbicide screening assay

In order to get idea about resistance level of collected populations, initial screening was carried out. The populations were chosen keeping in view the availability of seeds for further experiments. Fifteen populations were tested with ALS and ACCase inhibitors at a rate known to provide full control of susceptible *L. multiflorum* plants grown in pots (2 g a.i. ha^−1^ iodosulfuron-methyl sodium (≈0.0013%), 25 g a.i. ha^−1^ clodinafop-propargyl (≈0.0167%). For these purpose three trays of each population were prepared, one serving as control while the other two trays were treated with iodosulfuron-methyl sodium and clodinafop-propargyl. Plants were grown in a potting mixture consisting of soil, peat, and sand (2:1:1 w/w) containing all necessary macro- and micronutrients. After seedling emergence in a glass-house, trays were placed outdoor and thinned to one plant per hole in the tray. In total 104 individual plants per tray were maintained except for population ID-290 with only 45 individual plants due to a low germination. The herbicide efficacies were expressed as percentage survival and percentage decrease in fresh weight compared to control 4 weeks after herbicide application (Table 1).

**Table 1:** **Screening of *L. multiflorum* population using an ALS (iodosulfuron-methyl-sodium) and an ACCase (clodinafop-propargyl) inhibitor herbicide**.

**Populations**	**Herbicides**			
	**Iodosulfuron-methyl-sodium (2 g a.i. ha^−1^)**		**Clodinafop-propargyl (25 g a.i. ha^−1^)**	
	**Survival %**	**Percent decrease in FW**	**Survival %**	**Percent decrease in FW**
ID-290^$^	28.6	94.4	45.5	88.6
ID-06	69.2	71.9	94.3	32.7
ID-974	85.6	56.3	80.8	64.3
ID-775	86.5	50.1	83.7	53.4
ID-23	87.5	39.7	78.9	42.7
ID-691	87.5	66.6	91.4	47.8
ID-905	89.4	58.8	83.7	62.8
ID-01	91.4	54.5	72.1	68.0
ID-02	91.4	42.3	71.2	56.2
ID-904	93.3	45.8	77.9	54.6
ID-973	94.2	46.7	76.0	55.6
ID-774	95.2	47.4	74.0	63.5
ID-1023	96.2	9.9	75.0	54.4
ID-903	96.2	20.5	88.5	24.4
ID-906	96.2	50.2	76.9	58.6
ID-04	97.1	29.5	60.6	67.0

$ *Represent reference susceptible population. FW, Fresh weights*.

### Herbicide dose response study

Keeping in view initial screening and previous bioassays conducted in our laboratory, 17 populations were included in the dose-response studies. Due to low germination of the reference population ID-290, the commercial variety Sikem was also included as reference control. Seeds were sown in 2 L pots filled with the potting mixture used for the herbicide screening trials. Pots were placed in a glasshouse and sub-irrigated automatically one to three times per day depending on plant size. After germination the number of seedlings per pot was maintained at six and experiment was laid out in a completely randomized block design with three replications. Statistical analysis were done using R statistical software (R Development Team, http://www.r-project.org) with add on package *drc* (Ritz and Streibig, 2005; Knezevic et al., 2007) and a three-parameter log-logistic model were fitted to the data applying the *drc modelFit* function:

$$\begin{array}{l} Y=\frac{D}{1+\exp\left[b\left(\log\left(x\right)-\log\left(ED50\right)\right)\right]} \end{array}$$

where *Y* is the fresh weight, *x* represents herbicide dosage (g a.i. ha^−1^), *D* is mean response when herbicide dose is close to zero, *ED*_50_ is the dosage (g a.i. ha^−1^) that reduces fresh weight by 50% and *b* is the slope of the curve around ED_50_. The ED_50_ values for each population and herbicide are shown in Table 2 along with a resistance index (RI), which is calculated as the ratio between the estimated ED_50_ value of the tested and susceptible populations (ED_50*R*_/ED_50*S*_).

**Table 2:** **Dose response study of *L. multiflorum* populations using an ALS (iodosulfuron-methyl-sodium) and an ACCase (clodinafop-propargyl) inhibitor herbicide**.

**Populations**	**Herbicides**			
	**Iodosulfuron-methyl-sodium**		**Clodinafop-propargyl**	
	**ED_50_ (g a.i. ha^−1^)**	**RI**	**ED_50_ (g a.i. ha^−1^)**	**RI**
ID-290^$^	0.1 ± 0.02	0.4	nd	nd
Sikem^$^	0.3 ± 0.03	1.0	7.5 ± 1.1	1.0
ID-691	0.4 ± 0.1	1.4	nd	nd
ID-774	0.4 ± 0.1	1.5	4.8 ± 2.4	0.7
ID-775	0.4 ± 0.1	1.6	11.0 ± 2.6	1.5
ID-906	0.4 ± 0.1	1.6	10.5 ± 2.9	1.4
ID-905	0.5 ± 0.1	1.7	11.9 ± 1.5	1.6
ID-973	0.5 ± 0.1	1.7	8.2 ± 1.8	1.1
ID-01	0.5 ± 0.1	1.7	10.5 ± 2.1	1.4
ID-23	0.5 ± 0.12	1.7	nd	nd
ID-974	0.5 ± 0.09	1.9	12.0 ± 1.8	1.6
ID-06	0.6 ± 0.1	2.3	29.0 ± 4.6	3.9
ID-904	0.9 ± 0.1	3.3	9.4 ± 2.0	1.3
ID-20	1.0 ± 0.2	3.7	16.8 ± 4.2	2.3
ID-04	1.7 ± 0.2	6.4	nd	nd
ID-27^§^	9.0 ± 1.2	33.3	nd	nd
ID-07	>12.8^a^	>47.4	10.7 ± 2.9	1.4
ID-1023	>12.8^a^	>47.4	5.8 ± 1.3	0.8

Three parameter log logistic model was applied using add on package drc in R to calculate ED_50_. ED_50_ is the dosage (g a.i. ha^−1^) that reduced fresh weight by 50%. Resistance Index (RI) is calculated as the ratio between the estimated ED_50_ value of the tested and susceptible populations (ED_50R_/ED_50S_).

a Not possible to estimate the ED_50_ as plant fresh weight reduction was lower than 50% for all applied doses. nd, not determined due to low seed germination,

$ reference susceptible populations,

§ *metabolic resistant L. multiflorum from France*.

### Genotyping of ALS and ACCase genes from *L. multiflorum*

Leaf materials from all population were harvested for DNA extraction. Briefly, genomic DNA was extracted from plants using a DNeasy Plant Kit (Qiagen, USA). PCR was performed using the universal primers of ACCase genes as described by Délye et al. (2011) and specific primers of ALS gene of *L. multiflorum* from the GenBank accession number AF310684.1 (Table S1). PCR products of respective genes were amplified using initial denaturing for 5 min at 94°C, followed by 35 cycles consisting of 94°C for 40 s, 62°C for 35 s, 72°C for 30 s, and 72°C for 5 min for final extension. The PCR products were purified, sequenced and analyzed for single nucleotide polymorphism (SNP) using CLC main workbench according to manufacturer instructions (CLC bio, Aarhus, Denmark).

### RNA extraction and real time PCR

Total RNA was extracted from 50 mg of leaf material of individual plants of six selected populations using RNeasy Plant Mini Kit (Qiagen, Stanford, California, USA). RNA was extracted from three independent biological samples of selected populations. To eliminate genomic DNA from RNA samples they were treated twice with RNase-Free DNase (Qiagen, USA). Concentration and purity of total RNA were measured through NanoDrop 1000 spectrophotometer (Thermo Scientific, USA). Samples with concentration more than 100 ng μL^−1^ and optical density absorption ratios of A_260_/A_280_ and A_260_/A_230_ between 1.8–2.2 and 2.0–2.2, respectively, were taken for cDNA synthesis. For cDNA synthesis and differential gene expression we followed the guidelines proposed by Bustin et al. (2010). The qPCR reactions were performed using the StepOnePlus™ Real-Time PCR System Thermal Cycling Block (Applied Biosystems Foster City, USA). Two internal control genes were chosen based on their stable expression to herbicide treatment as found by Gaines et al. (2014). Same gene specific primers as described by Gaines et al. (2014) were used and presented in Table S2. Moreover, amplicon sizes were checked on 2.5% (w/v) agrose gels. Reactions were done in duplicate and a negative control consisting of template without primers were also included for each primer. Reactions were also conducted as described by Gaines et al. (2014) with some modification. Briefly, 20-μL volume of reaction included 10 μL of SyberGreen Master Mix, 2 μL of 1:10 diluted cDNA, 5 μL of 0.5 p mol μL^−1^ primers (1:1 mix of forward and reverse primers), and 3 μL of nuclease-free distilled water. Reaction conditions included 15 min incubation at 95°C, then 40 cycles of 95°C for 30 s and 60°C for 1 min, followed by a melt-curve analysis to confirm single PCR product amplification. Threshold-cycle (ΔC_T_) values were calculated for each reaction. Gene-specific PCR efficiency was used to calculate the expression of target genes relative to the expression of internal reference genes. Equivalent slopes for target and internal control genes were observed in amplification plots. The ΔC_T_ value was calculated as follows: ΔC_T_ (target genes) = C_T_ (target gene) − C_T_ (reference gene), where C_T_ is the cycle number at which PCR product exceeded a set threshold. Relative transcript level (RTL) was calculated through = 1 × 2^−Δ*CT*^. Transcript levels were calculated from three independent biological samples using three technical replicates of each biological sample.

### Orthologs of herbicide metabolism related genes and reference control genes in *A. thaliana* and *O. sativa*

The orthologs of HMR genes were identified in *A. thaliana* and *O. sativa*. The names of these orthologous are glycosyl-transferase (GT, At2g15490), nitronate monooxygenase (NMO, At5g64250), cytochrome P450 (CYP72A-1, At3g14680), and cytochrome P450 (CYP72A-2, At3g14690) with accession numbers NM_127109, NM_180932, NM_112329 and NM_112330 respectively, in *A. thalina*. Orthologs of HMR genes in the *O. sativa* were identified as Os01g0638600 (GT), Os08g0485400 (NMO), Os01g0627500 (CYP72A-1), and Os01g0728300 (CYP72A-2) with accession numbers NM_001050207, NM_001068622, NM_001050167, and NM_001050664, respectively. Rab GTPase (RGTP) and isocitrate dehydrogenase (IDE) were used as internal reference genes and identified as Os01g0276100 (NM_001049260) and Os01g0558600 (NM_001049871) in *O. sativa*, and At5G03290 (NM_120407) and At5G47200 (NM_124091) in *A. thaliana*. All the identified accession numbers of HMR orthologs were analyzed manually and validated through BLAST search using NCBI database (http://blast.ncbi.nlm.nih.gov/Blast.cgi).

### *In silico* analysis of expression pattern of herbicide metabolism related orthologs of *A. thaliana*

The behaviors of HMR genes under various experimental conditions and at different developmental stages were explored using the Genevestigator (http://www.genevestigator.com). The *Genevestigator* is a manually curated and well-annotated database of expression profiling from 11 different plant species with more than 26,000 exclusive plant samples (www.genevestigator.com, Feb. 2016). *In silico* analysis of the expression of identified transcription factors (TFs) was also explored using the condition tool of this database.

### *Cis* motif identification and occurrence of transcription factors in the promoters of herbicide metabolism related orthologous

The 1 kb upstream sequences from translational initiation codon of HMR orthologous in *A. thaliana* and *O. sativa* were obtained. In order to check occurrence of transcription factor (TF) in the promoter region of HMR genes, the promoters of HMR orthologous were analyzed using the web-based tool “The Plant Promoter Analysis Navigator (*PlantPAN 2.0*; http://PlantPAN2.itps.ncku.edu.tw).” This tool provides resources for detecting corresponding transcription factors and other regulatory elements in a promoter of a gene (Chow et al., 2015). The analysis was carried out separately on the promoters of each HMR orthologous of *A. thaliana* and *O. sativa* using Promoter analysis function of PlantPAN 2.0. This function recognizes the combinatorial *cis*-regulatory elements and associates them with corresponding transcription factors (TFs). Venn diagrams were plotted using online tools Venny 2.0 http://bioinfogp.cnb.csic.es/tools/venny/. All identified TFs were analyzed for biological functions using the AgriGO through singular enrichment analysis tool (http://bioinfo.cau.edu.cn/agriGO/). This analysis was performed with FDR correction and Fisher's exact test <0.05. Furthermore, identified TFs in *A. thaliana* and *O. sativa* were analyzed to find common and conserve TFs in both species. The InterPRO analysis was carried out to find conserve domain in TFs exclusively linked to the promoters of HMR genes.

The overrepresented *cis*-motif consensus patterns were identified using the Multiple Expectation maximization for Motif Elicitation (MEME) analysis tool (Bailey et al., 2006). The MEME was used to search best 5 *cis*-motif based on *E*-value, consensus patterns of 6–50 bases width, only on the forward strand of the input sequences and the distribution model used was the default Zero Or One Per Sequence (ZOOPS). A motif is a sequence pattern that occurs repeatedly in a group of DNA sequences. The motifs identified using MEME were analyzed through GOMO (Gene Ontology for Motifs; Buske et al., 2010). The purpose of GOMO is to identify possible roles (Gene Ontology terms) for DNA binding motifs. GOMO takes a motif and determines which GO terms are associated with the (putative) target genes of the binding motif. GOMO was run using the “multiple species category” which gave access to the plant database with significant threshold *q* < 0.01 and the number of score shuffling rounds over 1000.

## Results

### Herbicide screening assay

In our primary screening assay, all tested populations showed higher tolerance to either one or both of the herbicides compared to our reference susceptible population (ID-290). However, we found different levels of resistance among the tested populations. For example, population ID-1023 was the most resistant population to iodosulfuron-methyl-sodium with a survival percentage of 96.2% and decrease in fresh weight of only 9.9% (Table 1). Population ID-903 also had a high survival rate (96.2%) and exhibited a low decrease in fresh weight (20.5%) following iodosulfuron-methyl-sodium exposure. The most resistant populations to clodinofop-propargyl were ID-903 and ID-06 with 88.5 and 94.3% survival, and 24.4 and 32.7% decrease in their fresh weights, respectively, while ID-1023 was found to be moderately resistant with 75% survival and 54.4% decrease in fresh weight (Table 1). Other populations had a very interesting resistance pattern to both herbicides such as ID-04, which had 97.1% survival and 29.5% decrease in fresh weight after exposure to iodosulfuron-methyl-sodium, whereas clodinofop-propargyl showed 60.6% survival and 67% decrease in fresh weight. In order to obtain a deeper insight into the resistance pattern of the populations, a dose response experiment was carried out.

### Dose response study

The susceptible reference populations (ID-290 and Sikem) were found to be susceptible to both herbicides. Populations ID-1023 and ID-07 were found to be highly resistant to iodosulfuron-methyl-sodium with a RI of more than 47.4 (Table 2). The exact ED_50_ of ID-1023 and ID-07 could not be estimated, as fresh weight was higher than 50% over the whole dose ranges. The populations ID-06, ID-904, ID-20, and ID-04 exhibited 2.3, 3.3, 3.7, and 6.4 fold higher tolerance, respectively compared with the susceptible reference (Table 2).

In contrast to iodosulfuron-methyl-sodium, ID-1023 was susceptible to clodinafop-propargyl and the resistance index of ID-07 was only 1.4-fold higher than the standard reference used in this study. The populations ID-974, ID-20, and ID-06 showed 1.6, 2.3, and 3.9 fold higher tolerance, respectively (Table 2). Resistance index of ID-23, ID-04, ID-691, and ID-27 to clodinafop-propargyl were not determined due to low seed germination. The population ID-27 was obtained from France and found resistant to ACCase herbicides in our earlier bioassays (data not shown). ID-06 and ID-20 were moderately resistant with RIs of 3.9 and 2.3 to clodinafop-propargyl (Table 2).

### Genotyping to detect mutation in ALS and ACCase alleles of various populations

Comparison of the PCR fragments of the ALS gene from various *L. multiflorum* populations revealed a single nucleotide change from TGG to TTG at position 574 in 78 and 89% individuals of populations ID-1023 and ID-903, respectively (Table 3). This change leads to substitution of a tryptophan to leucine at 574 (Trp-574-Leu) and previous studies suggest that this mutation results in resistance to all classes of ALS inhibitors (Bernasconi et al., 1995; Bi et al., 2016).

**Table 3:** **Genotyping of various *L. multiflorum* populations to detect specific point mutations in ALS (= no mutation)**.

**Populations**	**Number of plants**	**Mutations analyzed**					
		**Pro-197 CCY**	**Ala-205 GCY**	**Asp-376 GAT, GAC**	**Arg-377 CGC**	**Trp-574 TGG**	**Ser-653 TCY**
ID-1023	9	=	=	=	=	**TTG**	=
ID-290	6	=	=	=	=	=	=
ID-691	6	=	=	=	=	=	=
ID-774	6	=	=	=	=	=	=
ID-775	6	=	=	=	=	=	=
ID-904	6	=	=	=	=	=	=
ID-905	6	=	=	=	=	=	=
ID-906	6	=	=	=	=	=	=
ID-973	6	=	=	=	=	=	=
ID-974	6	=	=	=	=	=	=
Sikem	6	=	=	=	=	=	=
ID-01	9	=	=	=	=	=	=
ID-04	9	=	=	=	=	=	=
ID-06	9	=	=	=	=	=	=
ID-07	9	=	=	=	=	=	=
ID-20	6	=	=	=	=	=	=
ID-23	6	=	=	=	=	=	=
ID-27	6	=	=	=	=	=	=
ID-903	9	=	=	=	=	**TTG**	=

*Letter shown in red and bold are identified mutations, and represent amino acids, TTG = Leucine*.

PCR fragments of the ACCase gene also revealed a single nucleotide change from ATT to AAT leading to substitution of isoleucine to asparagine at 2041 (Ile-2041-Asn) in one population ID-903 (Table 4). This Ile-2041-Asn substitution was reported to cause resistance to ACCase inhibitors (Délye et al., 2003, 2005; Martins et al., 2014).

**Table 4:** **Genotyping of various *L. multiflorum* populations to detect specific point mutations in ACCase (= no mutation)**.

**Populations**	**Number of plants**	**Mutations analyzed**						
		**Iso-1781 ATA**	**Trp-1999 TGG**	**Trp-2027 TGG**	**Iso-2041 ATT**	**Asp-2078 GAT**	**Cys-2088 TGC**	**Gly-2096 GGG**
ID-1023	9	=	=	=	=	=	=	=
ID-290	6	=	=	=	=	=	=	=
ID-691	6	=	=	=	=	=	=	=
ID-774	6	=	=	=	=	=	=	=
ID-775	6	=	=	=	=	=	=	=
ID-904	6	=	=	=	=	=	**TGT**	**GGA**
ID-905	6	=	=	=	=	=	**TGT**	**GGA**
ID-906	6	=	=	=	=	=	=	=
ID-973	6	=	=	=	=	=	=	=
ID-974	6	=	=	=	=	=	=	=
Sikem	6	=	=	=	=	=	=	=
ID-01	9	=	=	=	=	=	=	=
ID-04	9	=	=	=	=	=	=	=
ID-06	9	=	=	=	=	=	=	=
ID-07	9	=	=	=	=	=	=	=
ID-20	6	=	=	=	=	=	=	=
ID-23	6	=	=	=	=	=	=	=
ID-27	6	=	=	=	=	=	=	=
ID-903	9	=	=	=	**AAT**	=	=	=

*Letter shown in red and bold are identified mutations, and represent amino acids, AAT = Asn. Letters shown in black represent synonymous substitution*.

### Gene expression analysis of herbicide metabolism related genes

The expression patterns of four herbicide metabolism genes were tested in six populations of *L. multiflorum* without any herbicide exposure. These populations were selected based on their resistance patterns to ALS and ACCase inhibitors. Among the selected populations two (ID-23 and Sikem) were susceptible, two (ID-1023, ID-07) were resistant to ALS inhibitors, one (ID-27) was resistant to both ALS and ACCase inhibitors and one (ID-20) was found to be moderately resistant to both inhibitors. Before expression analysis, individual plants of these populations were tested for the presence of ALS and ACCase target site mutations. This revealed the presence of a mutation Trp-574-Leu in population ID-1023. In qPCR analysis, all four genes were exhibiting higher expression in some plants of the populations ID-20, ID-27, and ID-07. For example ID-20-3, ID-27-3, and ID-07-1 showed significant over-expression of all four genes (Table 5). ID-27-3 exhibited the highest expression of NMO, GT, and P450 among all populations. NMO was also highly expressed in all individual plants of population ID-27 and ID-07 (Table 5). However, there is considerable variation in the expression of GT, P450s, and NMO in populations and even in individuals of the same populations (Table 5). For example NMO was highly expressed in ID-20-3, but less expressed in the other plants of the same population, whereas expression of NMO was significant increased in all plants of ID-27, but P450s and GT were not significantly altered in ID-27-1. Nonetheless, some individuals of the three populations (ID-20, ID-27, and ID-07) exhibited significant constituent higher expression of HMR genes compared to the susceptible population (Table 5). Based on these results, we can conclude that populations ID-20, ID-27, and ID-07 showed signs of evolution of metabolic based herbicide resistance.

**Table 5:** **Expression of herbicide metabolism genes in selected populations of ***Lolium multiflorum*****.

**Population**	**Plant**	**Gene expression (Relative transcript level)**				
		**NMO**	**GT**	**CYP72A-1**	**CYP72A-2**	**IDE**
ID-20	ID-20-1	0.7 ± 0.04	1.3 ± 0.04	0.8 ± 0.03	0.8 ± 0.03	1.1 ± 0.09
	ID20-2	1.7 ± 0.33	0.9 ± 0.03	0.9 ± 0.02	1.2 ± 0.03	1.0 ± 0.03
	ID-20-3	4.8 ± 0.03^**^	3.5 ± 0.01^**^	1.9 ± 0.07^*^	1.7 ± 0.03^*^	1.0 ± 0.01
ID-23	ID-23-1	1.2 ± 0.03	1.0 ± 0.03	1.1 ± 0.04	1.5 ± 0.04	1.1 ± 0.03
	ID-23-2	0.6 ± 0.03	1.7 ± 0.03^*^	1.1 ± 0.01	1.8 ± 0.09^*^	1.1 ± 0.07
	ID-23-3	1.4 ± 0.03	1.1 ± 0.01	1.2 ± 0.03	0.8 ± 0.07	1.0 ± 0.00
ID-27	ID-27-1	4.3 ± 0.31^**^	1.3 ± 0.03	1.5 ± 0.02	1.4 ± 0.03	1.1 ± 0.04
	ID-27-2	4.9 ± 0.13^**^	1.8 ± 0.01^*^	1.3 ± 0.03	1.8 ± 0.07^*^	1.0 ± 0.01
	ID-27-3	14.8 ± 0.09^***^	4.0 ± 0.33^**^	4.5 ± 0.03^**^	3.4 ± 0.02^**^	1.1 ± 0.07
ID-07	ID-07-1	3.1 ± 0.29^*^	1.6 ± 0.03^*^	1.7 ± 0.03^*^	2.0 ± 0.03^*^	1.1 ± 0.03
	ID-07-2	3.0 ± 0.33^*^	1.7 ± 0.09^*^	1.4 ± 0.03	1.7 ± 0.03^*^	1.0 ± 0.00
	ID-07-3	2.4 ± 0.01^*^	1.6 ± 0.00^*^	1.4 ± 0.03	1.5 ± 0.03	1.0 ± 0.00
ID-1023	ID-1023-1	1.5 ± 0.03	0.8 ± 0.03	1.0 ± 0.03	1.5 ± 0.03	1.0 ± 0.07
	ID-1023-2	1.3 ± 0.13	1.1 ± 0.03	1.0 ± 0.03	1.5 ± 0.03	1.1 ± 0.05
	ID-1023-3	1.3 ± 0.03	1.5 ± 0.03	1.3 ± 0.00	1.4 ± 0.00	1.0 ± 0.03

Transcript levels were assessed in 3 weeks old plants. Relative transcript levels were based on reference susceptible population and internal reference genes. Sikem were used as reference control population and Rab GTPase (RGTP) gene was used as internal reference gene. The isocitrate dehydrogenase (IDE) was another reference control gene used in this study. The significance levels were calculated among three independent clones as biological sample of an individual plant (n = 3)

*, **, *** *indicate different level of significance calculated among plants of specified population using Tukey's test (P < 0.05, P < 0.01, P < 0.001)*.

### *In silico* analysis of expression pattern of herbicide metabolism related ortholog genes in *A. thaliana*

**Figure 1:**
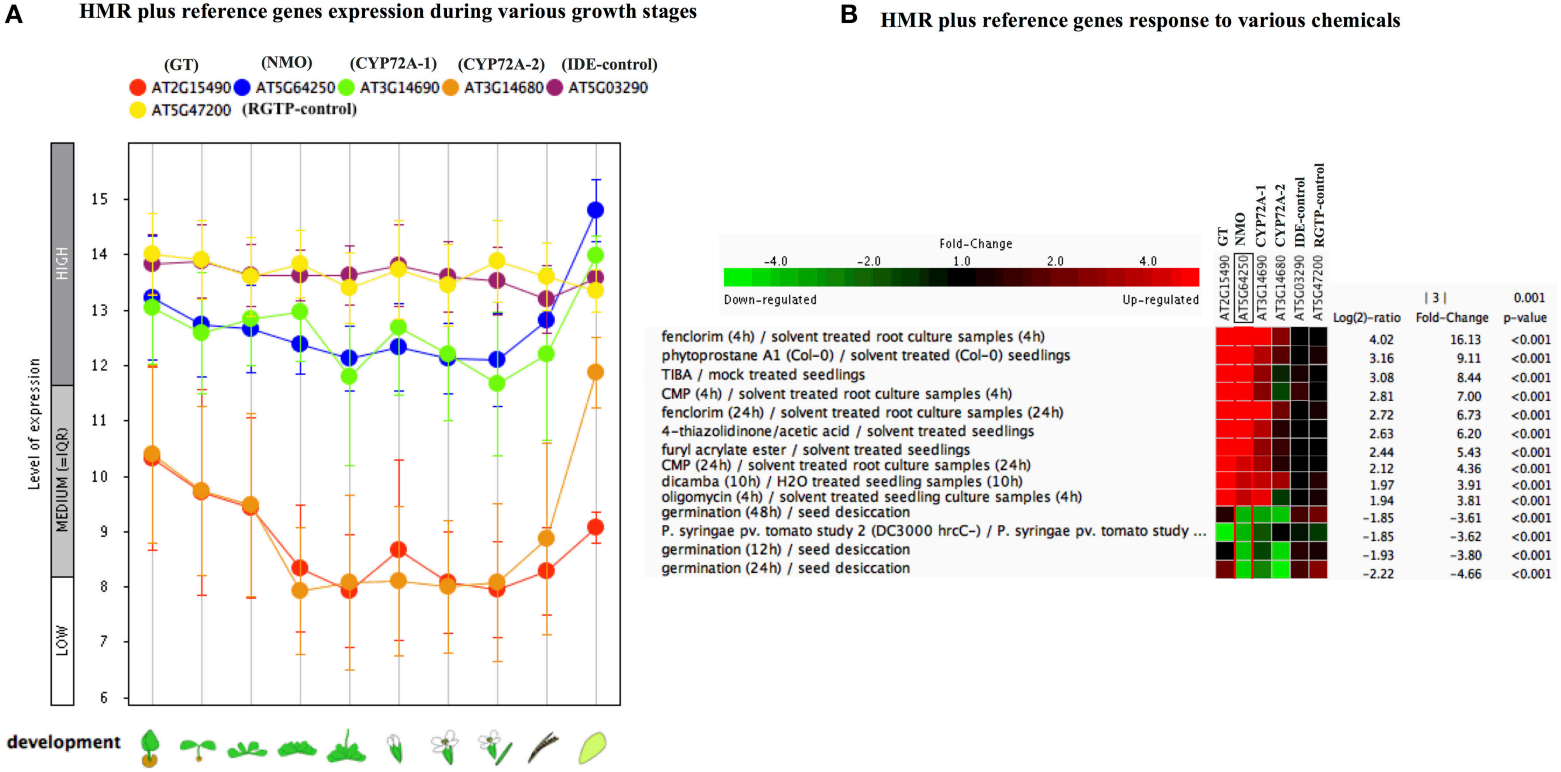
**Gene expression pattern of *Lolium multiflorum* orthologs of GT, NMO, CYPs along with control genes in *A. thaliana*. (A)** Developmental stage-specific expression pattern. Left to right, “germinating seed,” “seedling,” “young rosette,” “developed rosette,” “bolting rosette,” “young flower,” “developed flower,” “flower and silique,” “mature silique,” and “senescence.” Our selected genes were tending to show higher expression in senescence. “HIGH,” “MEDIUM,” and “LOW” expression were based on microarray gene expression data found in GENEVESTIGATOR (http://www.genevestigator.com). **(B)** Heat map of expression of selected genes in response to various external conditions such as chemicals were analyzed using Genevestigator perturbation tool. Relative expression of the genes was represented in log_2_ ratio and significant change in expression were filtered out based on NMO, *p* < 0.001 and fold change >3. Expression of HMR genes strongly induced in response to various chemicals including herbicides and herbicide safeners.

In order to check the behavior of HMR genes under various stresses, we utilized the publicly available gene expression database (Genevestigator). We checked the differential expression of HMR genes during different stages of plant development. NMO and CYP72A-1 had similar and high level of expression throughout developmental stages with maximum expression at senescence (Figure 1A). GT and CYP72A-2 had medium to low expression during different developmental stages except senescence, where CYP72A-2 tended toward higher expression (Figure 1A). Reference genes used in the study consistently showed a very high and stable expression pattern during different growth stages pointing them as good internal reference genes. In order to check behavior of these genes under various stress conditions, *in silico* expression was performed using the condition tool of the Genevestigator. This analysis revealed that 34 different perturbations (out of 3283 available in database) cause significant change in the expression (*p* < 0.001 and fold change >3.00) and the expression was most pronounced under various chemicals stress (Figure S1, Figure 1B). The strongest response was seen for the herbicide safener fenclorim, where expression increased 16-fold for NMO, 114-fold for GT, 9-fold for CYP72A-1, and 2.5-fold for CYP72A-2. Similarly herbicides such as dicamba and plant secondary metabolites phytoprostane strongly induce expression of these genes, whereas our control genes remain unaffected (Figure 1B). These results suggest that these herbicide metabolism genes have a role to detoxify herbicides and validate our observations *in L. multiflorum*.

### *Cis* motif identification and occurrence of transcription factors in the promoters

Discovery of regulatory *cis*-elements in the promoter regions is essential to understand the spatial and temporal expression pattern of involved genes in a specific function. These genes may be regulated by a common set of transcription factors, and can be detected by the occurrence of specific *cis*-regulatory motifs in the promoter region. Hence we analyzed the promoters regions of HMR genes and reported top five motifs common in both *A. thaliana* and *O. sativa*. Among the top five common motifs present in the investigated promoters of both plants, only the first motif was significant based on *E*-value (Table 6). In GOMO analysis, this motif was found to be involved in making CUL4-RING ubiquitin ligase complex with sigma factor activity and localized in chloroplast and mitochondrial inner membrane. Cullin-RING (CUL) complexes represent a predominant group of ubiquitin E3 ligases with vital role in coping with environmental stresses and maintaining genome stability (Guo et al., 2013). Remaining top four common motifs were not considered significant based on *E*-value. However, these were all involved in transcription factor activity, helicase activity, regulation of transcription, kinase activity and circadian rhythm (Table 6).

**Table 6:** **Motifs found in of promoter of NMO, CYPs, and GT in the MEME analysis**.

**MEME Identified Motif logo**	**Consensus motif in HMR**	**Organism**	***E*-value**	**Number of GO term identified by GOMO**	**Role of motif identify by GOMO**
**1-	CCGCATGCATATGTTCTCCAANNNTTTCAGTTTTNTCTGCC	AT & OS	2.3e-002	05	CC chloroplast
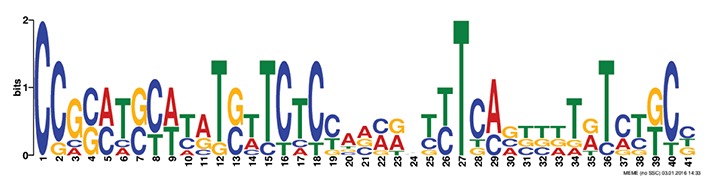					CC CUL4 RING ubiquitin ligase complex
					MF sigma factor activity
					CC respiratory chain complex I
					CC mitochondrial inner membrane
2-	GAAGGGTATGCNNGGNNTGGANTNNGGCNNNGGGCAAGNGC	AT & OS	4.0e-001	03	CC mitochondrion
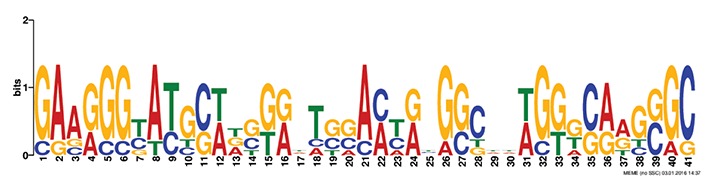					MF helicase activity
					CC chloroplast envelope
3-	TNNNTACCTCTNTCTTTNTCTNCACTCTTAGAAA	AT & OS	2.2e+003	10	CC nucleus
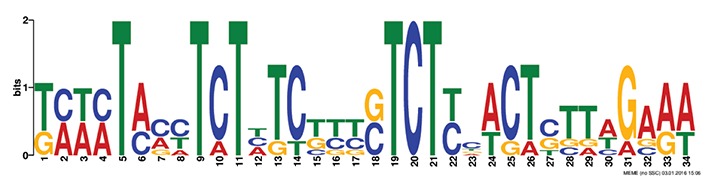					CC plasma membrane
					BP regulation of transcription, DNA-dependent
					BP carpel development
					BP circadian rhythm
					BP regulation of transcription
					MF transcription factor activity
					MF protein binding
					MF DNA binding
					MF nucleic acid binding
4-	ACTTCTCTACCTCTNTCTCTCTCTTCNCT	AT & OS	7.1e+003	14	MF transcription factor activity
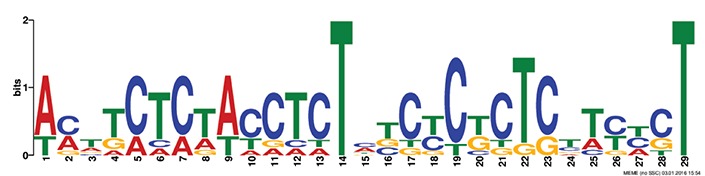					MF protein serine/threonine kinase activity
					MF DNA binding
					MF kinase activity
					MF protein binding
					CC nucleus
					CC plasma membrane
					BP regulation of transcription, DNA-dependent
					BP leaf development
					BP transmembrane receptor protein tyrosine kinase signaling pathway
					BP anther dehiscence
					BP regulation of transcription
					BP carpel developmen
5-	GGAGAGNG	AT & OS	3.1e+005	06	MF transcription factor activity
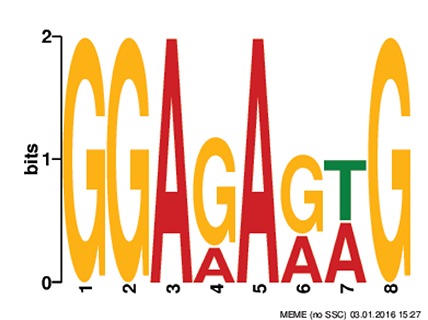					MF kinase activity
					CC nucleus
					CC chloroplast
					BP regulation of transcription, DNA-dependent
					CC plasma membrane

Top 5 common and exclusive motifs of A. thaliana and O. sativa identified by MEME in HMR promoters presented here with their E-value. The E-value is an estimate of the expected number of motifs with the given log likelihood ratio (or higher), and with the same width and site count, that one would find in a similarly sized set of random sequences (sequences where each position is independent and letters are chosen according to the background letter frequencies). GOMO is used to identify role associated to this DNA motif using the plant genomes as reference. GOMO provides information regarding the novelty of the motif and the probability that this motif is involved in transcription regulation. **Represent significant motif, AT = Arabidopsis thaliana; OS = Oryza sativa; CC = cellular component; MF = molecular function; BP = biological processes.

**Figure 2:**
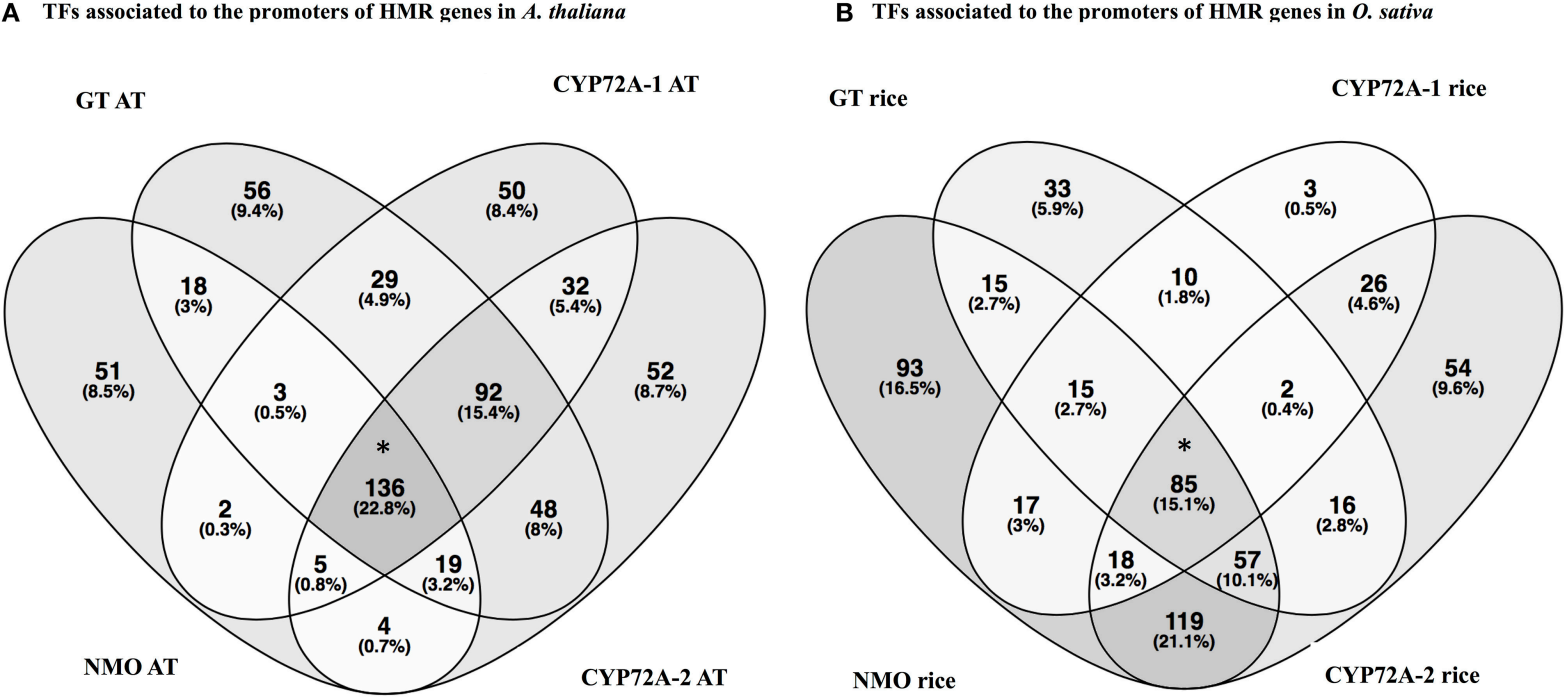
**Venn diagrams represent the transcription factors associated to the promoters of HMR genes of *A. thaliana* (A) and *O. sativa* (B)**. Analysis is done through The Plant Promoter Analysis Navigator (PlantPAN 2.0) using the promoter analysis function. **(A)** TFs associated to the promoters of HMR genes in *Arabidopsis thaliana*. **(B)** TFs associated to the promoters of HMR genes in *Oryza sativa*. ^*^TFs associated to all four HMR genes of *A. thaliana* and *O. sativa*. One hundred and thirty-six TFs commonly associated to promoters of all four MR genes in *A. thaliana* and 85 TFs linked to the promoters of HMR genes of *O. sativa*. AT represents *Arabidopsis thaliana*. Venn diagrams were plotted using online tool Venny 2.0 http://bioinfogp.cnb.csic.es/tools/venny.

In order to check the occurrence of TFs associated to the promoter regions of HMR genes, the *PlantPAN* promoter analysis tool was utilized. The analysis elucidate TFs associated to the promoters of individual gene and can determine common TFs among the four HMR genes. Interestingly, 136 TFs were common in all four HMR genes in *A. thaliana* and 85 TFs were found to be commonly associated to the promoters of HMR genes of *O. sativa* (Figure 2, Table S3). Among them, 14 TFs were conserved and exact homologs between *A. thaliana* and *O. sativa* (Table 7). In order to get deeper insights about these conserved TFs, their expression patterns were analyzed at different developmental stages and under various stress conditions. This revealed that two C2H2 type zinc finger transcription factors (ZAT6 and ZAT10) were up regulated in response to herbicides, whereas squamosa promoter-binding (SPB) type did not respond to chemicals (Figure S3). The expression of ZAT6 and ZAT10 is very similar to HMR genes during different growth developmental stages and under chemical stress (Figure S3). These zinc finger transcription factors had the highest expression level throughout developmental stages with top most expression at senescence and highly up regulated in response to herbicides and herbicide safeners (Figure S3).

**Table 7:** **Names of the conserved TFs to the promoters of HMR genes between *A. thaliana* and *O. sativa* associated**.

**S.no**.	***A. thaliana***	***O. sativa***	**Description**
1	AT4G34000	OS06G0211200	ABSCISIC ACID-INSENSITIVE 5-like protein 6
2	AT3G19290	OS09G0456200	ABSCISIC ACID-INSENSITIVE 5-like protein 7
3	AT1G25560	OS01G0693400	AP2/ERF and B3 domain-containing transcription repressor TEM1
4	AT1G18330	OS06G0728700	Homeodomain-like superfamily protein
5	AT1G27370	OS02G0139400	Squamosa promoter-binding-like protein 10
6	AT1G27360	OS06G0703500	Squamosa promoter-binding-like protein
7	AT1G20980	OS08G0513700	Squamosa promoter-binding-like protein
8	AT3G57920	OS06G0703500	Squamosa promoter-binding-like protein
9	AT1G76580	OS08G0513700	Squamosa promoter-binding-like protein
10	AT2G33810	OS04G0551500	Squamosa promoter-binding-like protein 3
11	AT5G18830	OS05G0408200	Squamosa promoter-binding-like protein 7
12	AT1G27730	OS12G0583700	ZAT10, salt tolerance zinc finger
13	AT5G04340	OS03G0437100	ZAT6, C2H2 zinc finger of *Arabidopsis thaliana* 6
14	AT5G43170	OS12G0583700	Zinc-finger protein

## Discussion

Herbicide resistance is an evolutionary process and a classic example of rapid dynamic adaptation to human mediated exerted selection pressure (Powles and Yu, 2010; Neve et al., 2014). Due to increasing incidences of herbicide resistance worldwide, a better understanding of resistance evolution and the basis of resistance mechanisms are imperative for adopting better weed control strategies. Initial screening and whole plant dose response bioassays are utilized to detect resistance level of our sampled populations. In the screening assay, ID-1023 and ID-903 were the most resistant populations to iodosulfuron-methyl-sodium, whereas ID-903 and ID-06 were the most resistant populations to clodinofop-propargyl. This bioassay indicates the presence of resistant populations in our sampled populations to both modes of action herbicides but in order to get a more detailed overview of the resistance level, a dose response study was carried out. ID-1023 was also found highly resistant to iodosulfuron-methyl-sodium in this bioassay, similar to previous screening bioassay. Population ID-07 was included in the dose response experiment and found highly resistant to iodosulfuron-methyl-sodium. Other populations ID-06, ID-904, ID-20, and ID-04 exhibited two to six-fold higher resistance index (RI) to iodosulfuron-methyl-sodium, respectively compared with the susceptible reference. However, collected populations were not highly resistant to clodinofop-propargyl. Populations ID-06 and ID-20 were moderately resistant with RIs of 3.9 and 2.3 to clodinafop-propargyl. Populations ID-1023 was found susceptible to clodinafop-propargyl in dose response bioassay. The ACCase resistant population (ID-27) was obtained from France and included in gene expression experiment. All populations were genotyped to elucidate ALS and ACCase TSR mutations. In *Lolium* spp., seven mutations at positions Iso-1781, Trp-1999, Trp-2027, Iso-2041, Asp-2078, Cys-2088, and Gly-2096 are known to confer TSR to ACCase (Malone et al., 2014; Martins et al., 2014). For ALS inhibitors, eight mutations in ALS at Ala-122, Pro-197, Ala-205, Asp-376, Arg-377, Trp-574, Ser-653, and Gly-654 have been reported to confer resistance (Han et al., 2012) and six substitutions at two positions (Pro-197 and Trp-574) were found in ALS of *Lolium* spp (Liu et al., 2014). In populations ID-1023 and ID-903, we identified a well-known Trp-574-Leu mutation in the ALS gene that can explain resistance to iodosulfuron-methyl-sodium. This mutation can cause resistance to all classes of ALS inhibitors (Bernasconi et al., 1995; Bi et al., 2016). Similarly, in population ID-903 a mutation Ile-2041-Asn in ACCase gene was identified. This mutation Ile-2041-Asn is known to confer ACCase resistance (Délye et al., 2003, 2005; Martins et al., 2014) and can explain resistance of ID-903 to clodinofop-propargyl. We did not find mutations in other collected populations and hence they might contain metabolic based resistance. In order to check metabolic based herbicide resistance, expression of four herbicide metabolism genes (two P450, GT, and NMO) was analyzed in six populations. All four genes were found to exhibit enhanced expression in individuals of the ID-27, ID-07, and ID-20 populations. These genes have been known to be constituently over-expressed in metabolic based resistant individuals of *L. rigidium* (Gaines et al., 2014). Based on our results, we can conclude that these populations have evolved metabolic based herbicide resistance or NTSR. Overall multiple herbicide resistances consisting of both TSR and NTSR mechanisms were identified in Danish *L. multiflorum* populations. The co-existence of one to several resistance mechanisms in herbicide resistant individuals adds complexity to resistance management. Metabolic based resistance is due to induced regulators and protectors which are triggered by herbicide stress (Délye, 2013) and depends on correct and timely regulation of genes that are responsible for reduction of herbicide efficiency. Our results revealed the constituent over-expression of a set of four genes in metabolic based resistant individuals of certain populations but the question is how constituent over-expression of these genes is coordinated and maintained for next generations in metabolic based herbicide resistant individuals? We speculate that specific transcription factors play such role for gene expression control. In order to identify transcription factors, we performed a detailed co-expression network and comprehensive bioinformatics analysis on the promoters region of these genes.

**Figure 3:**
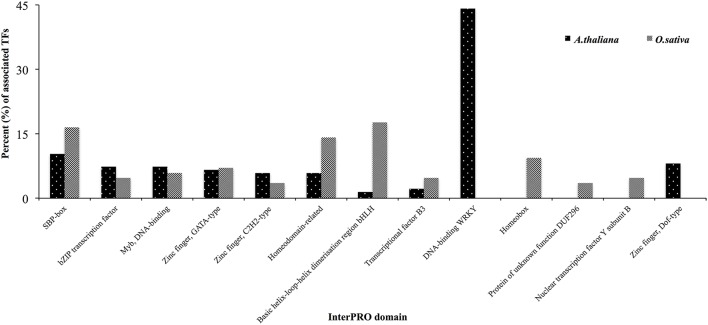
**Classification of TFs based on InterPRO analysis in *A. thaliana* and *O*. *sativa***. Percent of TFs associated to the promoters of all four HMR genes were presented.

One hundred and thirty-six TFs were common in all four HMR genes in *A. thalina* and 85 TFs were found to be commonly associated to the promoters of HMR genes of *O. sativa*. Functional *in silico* analysis revealed the significant involvement of these TFs in both *A. thalina* and *O. sativa* of various metabolic processes, which were regulated transcriptionally (Figure S2). Dividing TFs revealed that the percentage of squamosa promoter-binding (SPB-box), bZIP transcription factor, Myb DNA binding domains, zinc finger GATA type, and zinc finger C2H2 type transcription factors. Transcription factors containing these domains have a well-established role in abiotic stress tolerance (Dezar et al., 2005; Dubos et al., 2010; Cabello and Chan, 2012; Song et al., 2012; Zhang et al., 2014; Li et al., 2015; Wei et al., 2015). Interestingly WRKY like and Zinc finger dof-type transcription factors were exclusively found in *A. thaliana*. Contrary, homeobox and nuclear transcription factors Y subunit B were only found in *O. sativa*. The proportion of bHLH type and homeodomain related transcription factors were also higher in *O. sativa* compared to *A. thaliana* (Figure 3). This could be due to divergence in regulatory region of HMR genes between monocot and dicot species.

**Figure 4:**
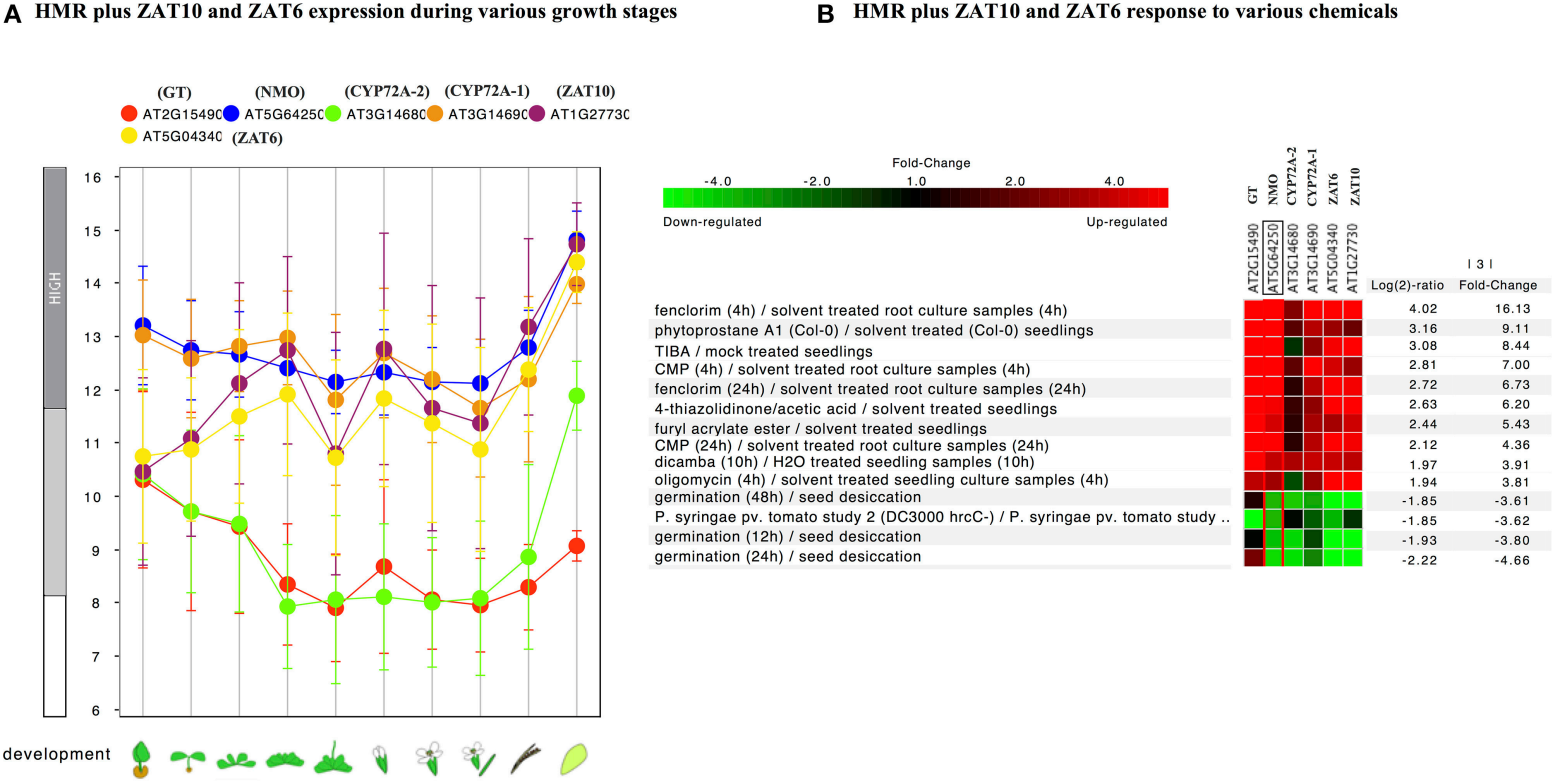
**Expression pattern of identified conserved transcription factors ZAT10 and ZAT6 genes along with of HMR. (A)** Developmental stage-specific expression pattern. Left to right, “germinating seed,” “seedling,” “young rosette,” “developed rosette,” “bolting rosette,” “young flower,” “developed flower,” “flower and silique,” “mature silique,” and “senescence.” Our identified TFs were tending to show higher expression in senescence similar to genes. “HIGH,” “MEDIUM,” and “LOW” expression were based on microarray gene expression data found in GENEVESTIGATOR (http://www.genevestigator.com). **(B)** Heat map of the expression of identified TFs along with HMR genes under various chemical stress were analyzed using Genevestigator perturbation tool. Relative expression of the genes was represented in log_2_ ratio and significant change in expression were filtered out based on NMO, *p* < 0.001 and fold change >3. Expression of these genes strongly induced in response to various chemicals including herbicides and herbicide safeners.

Further individual analysis of linked transcription factors to the promoters of both species revealed that 14 transcription factors were conserved between *A. thaliana* and *O. sativa*. These were squamosa promoter-binding (SPB-box) and zinc finger like transcription factors. The co-expression analysis revealed that zinc finger transcription factors, ZAT6 and ZAT10 were behaving similar to HMR genes in response to xenobiotics (Figure 4) and known to enhance tolerance of plants against abiotic stresses in previous studies (Mittler et al., 2006; Gourcilleau et al., 2011; Liu et al., 2013; Shi et al., 2014). In contrary SPB-box transcription factors did not alter their expression in response to xenobiotics. These SPB-box TFs are known to play role in plant reproductive growth and shaping plant life (Chen et al., 2010; Kim et al., 2012; Zhang and Li, 2013).

The efforts to identify *cis* regulatory elements in the promoter region of HMR genes of *A. thaliana* and *O. sativa* led us to the identification of a significant motif involved in making CUL4-RING ubiquitin ligase complex. This complex might help in correct and timely regulation of genes responsible in reduction of herbicide efficiency. The CUL4-RING ubiquitin ligase complex is known to play role in genome stability, chromatin dynamics, gene expression, and parental imprinting (Molinier et al., 2008; Zhang et al., 2008; Dumbliauskas et al., 2011; Pazhouhandeh et al., 2011; Guo et al., 2013).

However, experimental evidence is required to know about the role of the identified motifs and transcription factors in the regulation of HMR genes in herbicide stress environment. Response of knock out or over-expressed lines of these two transcription factors toward herbicides could be tested. Similarly, the role of identified motifs could be tested experimentally through deleting motifs in the promoter region of HMR genes to elucidate whether HMR genes are regulated at the transcription level or the post-transcriptional level or even through epigenetic processes.

## Conclusion

We have provided the resistance profile of *L. multiflorum* populations in Denmark and identified four populations resistant to acetolactate synthase (ALS) inhibitors and three resistant to acetyl-CoA carboxylase (ACCase) inhibitors. This resistance was due to presence of mutations Trp-574-Leu and Ile-2041-Asn in ALS and ACCase, respectively as well as constituent higher expression of metabolism related genes in individuals of resistant populations. Furthermore, application of bioinformatics tools such as in promoter analysis and co-expression network led us to the identification of a significant motif and two putative zinc finger transcription factors with probable role in herbicide resistance. In nutshell, we confirm the role of four putative genes earlier identified in *L. rigidum* by Gaines et al. (2014) in metabolic based herbicide resistance and insights about transcriptional regulation of metabolic based herbicide resistance. The identification of two putative zinc finger transcription factors and a significant motif is an important step forward toward a better understanding of metabolism-based herbicide that can be utilized to devise novel strategies of weed management in future.

## Author contributions

KM, SM, PK, and MK had designed this study. SM collected the field populations. KM prepared the material for greenhouse and laboratory experiments and carried out them. KM and SM prepared the statistical analysis of greenhouse experiments. SM and MK help in conducting bioassay experiments and molecular analysis, respectively. KM, SM, PK, and MK wrote the manuscript. SM, PK, and MK supervised the project.

### Conflict of interest statement

The authors declare that the research was conducted in the absence of any commercial or financial relationships that could be construed as a potential conflict of interest.

## Abbreviations

ALS: Acetolactate synthase
ACCase: Acetyl-CoA carboxylase
GTs: Glycosyl-transferases
GSTs: Glutathione *S*-transferases
HMR: Herbicide metabolism related
TSR: Target site resistance
NTSR: Non target site resistance
P450s: Cytochrome P450 monooxygenase
NMO: Nitronate monooxygenase
DNA: Deoxynucleic acid
TFs: Transcription factors
MEME: Multiple Expectation maximization for Motif Elicitation
qPCR: Quantitative polymerase chain reaction
RNA: Ribonucleic acid
RI: Resistance index.
