# Supplementary material for: Multiple Herbicide Resistance in Lolium multiflorum and Identification of Conserved Regulatory Elements of Herbicide Resistance Genes
Source: Front Plant Sci. 2016 Aug 5;7:1160. doi: 10.3389/fpls.2016.01160 (PMC4974277; doi:10.3389/fpls.2016.01160)
Supplement: Supplementary file 1 [file Table1.DOCX]

**Table S1:** Primers used for genotyping of ALS and ACCase

| **Primer** | **Gene** | **Sequence 5’ to 3’** |
| --- | --- | --- |
| ALS1F | ALS | CTCAATGGAGATCCACCAG |
| ALS1R | ALS | AGACCCATGAGGGTAGTTGTAA |
| ALS2F | ALS | CTCATGGGTCTTGGCAACTT |
| ALS2R | ALS | TGCCATCACCTTCCATGATA |
| ACcp1 | ACCase | CAACTCTGGTGCTIGGATIGGCA |
| ACcp1R | ACCase | GAACATAICTGAGCCACCTIAATATATT |
| ACcp4 | ACCase | CAGCITGATTCCCAIGAGCGITC |
| ACcp2R | ACCase | CCATGCAITCTTIGAGITCCTCTGA |
