# Supplementary material for: Multiple Herbicide Resistance in Lolium multiflorum and Identification of Conserved Regulatory Elements of Herbicide Resistance Genes
Source: Front Plant Sci. 2016 Aug 5;7:1160. doi: 10.3389/fpls.2016.01160 (PMC4974277; doi:10.3389/fpls.2016.01160)
Supplement: Supplementary file 2 [file Table2.DOCX]

**Table S2:** Real time PCR primers of HMR genes used for gene expression

| Gene | Primer | Sequence |
| --- | --- | --- |
| CYP72A1 | Forward | GATAAAGAGGATGCTGCCTGTGTTCGCTA |
|  | Reverse | CTGGGATAAAAATAGTCTGAAAAGCCATT |
| CYP72A2 | Forward | TTTCTTCGCTACAGGCAGAA |
|  | Reverse | CAAAGCACAGGAGAATCCTAAATCC |
| GST | Forward | TGTGTTTACACGAAAAGAGAGAGCTTA |
|  | Reverse | AGAGCAAAAGACTACGCCATTAAGG |
| NMO | Forward | TAGTGGATCTCGTTTCAGATAGTGCTAC |
|  | Reverse | ATACAGAGGATGTGCAAAGCTTTCC |
| IDE | Forward | GCGCAACATCATAACAGCAC |
|  | Reverse | GGTGAAGGTGGCATTTGTCT |
| RGTP | Forward | GATGTGACTGACCAAGAGAGCTTCA |
|  | Reverse | CTCAGCTAAGTCGCATTTGTTCCCC |
