# Supplementary material for: Multiple Herbicide Resistance in Lolium multiflorum and Identification of Conserved Regulatory Elements of Herbicide Resistance Genes
Source: Front Plant Sci. 2016 Aug 5;7:1160. doi: 10.3389/fpls.2016.01160 (PMC4974277; doi:10.3389/fpls.2016.01160)
Supplement: Supplementary file 3 [file Table3.DOCX]

**Table S3: Transcription factors associated to all four HMR genes in *A.thaliana* and *O.sativa***

| **S.NO** | ***Arabidopsis thanliana*** | | ***Oryza sativa*** | |
| --- | --- | --- | --- | --- |
|  | **Gene ID** | **Description** | **Gene ID** | **Description** |
| 1 | AT4G34000 | ABSCISIC ACID-INSENSITIVE 5-like protein 6 | OS06G0211200 | ABA-responsive element binding protein 1 (AREB1) |
| 2 | AT3G19290 | ABSCISIC ACID-INSENSITIVE 5-like protein 7 | OS02G0766700 | ABA-responsive element binding protein 2) (AREB2) |
| 3 | AT1G13260 | AP2/ERF and B3 domain-containing transcription factor RAV1 | OS01G0141000 | AP2/ERF and B3 domain-containing protein Os01g0141000 |
| 4 | AT1G25560 | AP2/ERF and B3 domain-containing transcription repressor TEM1 | OS01G0693400 | AP2/ERF and B3 domain-containing protein Os01g0693400 |
| 5 | AT1G23420 | Axial regulator YABBY 4 | OS01G0835600 | AT hook, DNA-binding, conserved site domain containing protein |
| 6 | AT3G54620 | basic leucine zipper 25 | OS08G0101000 | B3 domain-containing protein IDEF1 |
| 7 | AT1G68640 | bZIP transcription factor family protein | OS04G0676600 | B3 domain-containing protein |
| 8 | AT5G04340 | C2H2 zinc finger of Arabidopsis thaliana 6 | OS03G0728900 | bHLH DNA-binding domain containing protein |
| 9 | AT1G02030 | C2H2-like zinc finger protein | OS08G0487700 | bHLH DNA-binding domain containing protein |
| 10 | AT2G45120 | C2H2-like zinc finger protein | OS02G0705500 | bHLH domain containing protein |
| 11 | AT3G60580 | C2H2-like zinc finger protein | OS01G0915600 | bHLH transcription factor |
| 12 | AT3G49930 | C2HC zinc fingers superfamily protein | OS02G0564700 | bHLH transcription factor |
| 13 | AT1G29160 | Dof zinc finger protein DOF1.5 | OS03G0802900 | bHLH transcription factor |
| 14 | AT1G64620 | Dof zinc finger protein DOF1.8 | OS06G0193400 | bHLH transcription factor |
| 15 | AT2G37590 | Dof zinc finger protein DOF2.4 | OS07G0193800 | bHLH transcription factor |
| 16 | AT3G21270 | Dof zinc finger protein DOF3.1 | OS05G0103000 | bHLH transcription factor |
| 17 | AT3G45610 | Dof zinc finger protein DOF3.2 | OS04G0350700 | bHLH transcription factor 9 |
| 18 | AT3G47500 | Dof zinc finger protein DOF3.3 | OS09G0510500 | bHLH transcription factor 9 |
| 19 | AT4G38000 | Dof zinc finger protein DOF4.7 | OS05G0489700 | BZIP protein BZO2H3 |
| 20 | AT5G39660 | Dof zinc finger protein DOF5.2 | OS09G0456200 | BZIP transcription factor ABI5 |
| 21 | AT5G60200 | Dof zinc finger protein DOF5.3 | OS12G0583700 | C2H2-type domain containing protein |
| 22 | AT5G60850 | Dof zinc finger protein DOF5.4 | OS03G0437100 | C2H2-zinc finger domain containing protein |
| 23 | AT5G62940 | Dof zinc finger protein DOF5.6 | OS03G0437200 | C2H2-zinc finger domain containing protein |
| 24 | AT2G27050 | ETHYLENE INSENSITIVE 3-like 1 protein | OS02G0824300 | DUF296 domain containing protein |
| 25 | AT5G21120 | ETHYLENE INSENSITIVE 3-like 2 protein | OS08G0512400 | DUF296 domain containing protein |
| 26 | AT3G14230 | Ethylene-responsive transcription factor RAP2-2 | OS06G0326000 | DUF296 domain containing protein. |
| 27 | AT3G24050 | GATA transcription factor 1 | OS05G0578900 | GAGA-type zinc finger transcription factor |
| 28 | AT3G54810 | GATA transcription factor 10 | OS02G0220400 | GATA transcription factor 16 |
| 29 | AT3G06740 | GATA transcription factor 16 | OS03G0831200 | GATA transcription factor 19 |
| 30 | AT3G16870 | GATA transcription factor 17 | OS06G0571800 | GATA transcription factor 20 |
| 31 | AT2G45050 | GATA transcription factor 2 | OS01G0976800 | GATA zinc finger domain containing protein |
| 32 | AT1G51600 | GATA transcription factor 25 | OS05G0155400 | GATA zinc finger domain containing protein |
| 33 | AT3G21175 | GATA transcription factor 27 | OS03G0727000 | Homeobox protein knotted-1-like 6 |
| 34 | AT3G60530 | GATA transcription factor 4 | OS08G0187500 | Homeobox-leucine zipper protein ROC1 |
| 35 | AT4G16141 | GATA type zinc finger transcription factor family protein | OS04G0627000 | Homeobox-leucine zipper protein ROC2 |
| 36 | AT1G30490 | Homeobox-leucine zipper protein ATHB-9 | OS10G0575600 | Homeobox-leucine zipper protein ROC3 |
| 37 | AT1G01060 | Homeodomain-like superfamily protein | OS04G0569100 | Homeobox-leucine zipper protein ROC4 |
| 38 | AT1G18330 | Homeodomain-like superfamily protein | OS02G0674800 | Homeobox-leucine zipper protein ROC5 |
| 39 | AT3G10113 | Homeodomain-like superfamily protein | OS09G0526200 | Homeobox-leucine zipper protein ROC6 |
| 40 | AT5G16560 | Homeodomain-like superfamily protein | OS08G0136100 | Homeobox-leucine zipper protein ROC7 |
| 41 | AT5G37260 | Homeodomain-like superfamily protein | OS06G0728700 | Homeodomain-like containing protein |
| 42 | AT3G15500 | NAC domain-containing protein 55 | OS04G0580700 | MADS box transcription factor MADS17 |
| 43 | AT5G65100 | Putative ETHYLENE INSENSITIVE 3-like 5 protein | OS03G0122600 | MADS-box transcription factor 50 |
| 44 | AT2G47070 | Squamosa promoter-binding-like protein 1 | OS10G0536100 | MADS-box transcription factor 56 |
| 45 | AT1G27370 | Squamosa promoter-binding-like protein 10 | OS02G0682200 | MADS-box transcription factor 6 |
| 46 | AT1G27360 | Squamosa promoter-binding-like protein 11 | OS08G0531700 | MADS-box transcription factor 7 |
| 47 | AT3G60030 | Squamosa promoter-binding-like protein 12 | OS09G0507200 | MADS-box transcription factor 8 |
| 48 | AT1G20980 | Squamosa promoter-binding-like protein 14 | OS09G0538400 | Myb transcription factor |
| 49 | AT3G57920 | Squamosa promoter-binding-like protein 15 | OS11G0180900 | Myb transcription factor domain containing protein |
| 50 | AT1G76580 | Squamosa promoter-binding-like protein 16 | OS12G0175400 | Myb-like 2 transcription factor |
| 51 | AT5G43270 | Squamosa promoter-binding-like protein 2 | OS05G0429900 | Myb-like 4 DNA-binding domain |
| 52 | AT2G33810 | Squamosa promoter-binding-like protein 3 | OS03G0224200 | Myb-like DNA-binding domain |
| 53 | AT1G53160 | Squamosa promoter-binding-like protein 4 | OS02G0725900 | Nuclear transcription factor Y subunit B-1 |
| 54 | AT3G15270 | Squamosa promoter-binding-like protein 5 | OS01G0834400 | Nuclear transcription factor Y subunit B-2 |
| 55 | AT1G69170 | Squamosa promoter-binding-like protein 6 | OS05G0463800 | Nuclear transcription factor Y subunit B-3 |
| 56 | AT5G18830 | Squamosa promoter-binding-like protein 7 | OS05G0573500 | Nuclear transcription factor Y subunit B-4 |
| 57 | AT2G42200 | Squamosa promoter-binding-like protein 9 | OS07G0184800 | putative histone H1 |
| 58 | AT1G55520 | TATA-box-binding protein 2 | OS11G0602200 | SET domain protein SDG111 |
| 59 | AT4G14770 | TESMIN/TSO1-like CXC 2 | OS09G0501600 | Similar to MYC1 |
| 60 | AT5G08130 | Transcription factor BIM1 | OS01G0292900 | Squamosa promoter-binding-like protein 1 |
| 61 | AT1G32640 | Transcription factor MYC2 | OS06G0659100 | Squamosa promoter-binding-like protein 10 |
| 62 | AT5G65210 | Transcription factor TGA1 | OS06G0663500 | Squamosa promoter-binding-like protein 11 |
| 63 | AT5G06950 | Transcription factor TGA2 | OS06G0703500 | Squamosa promoter-binding-like protein 12 |
| 64 | AT5G10030 | Transcription factor TGA4 | OS08G0509600 | Squamosa promoter-binding-like protein 14 |
| 65 | AT5G06960 | Transcription factor TGA5 | OS08G0513700 | Squamosa promoter-binding-like protein 15 |
| 66 | AT3G12250 | Transcription factor TGA6 | OS08G0531600 | Squamosa promoter-binding-like protein 16 |
| 67 | AT1G77920 | Transcription factor TGA7 | OS09G0507100 | Squamosa promoter-binding-like protein 18 |
| 88 | AT3G16857 | Two-component response regulator ARR1 | OS01G0922600 | Squamosa promoter-binding-like protein 2 |
| 69 | AT4G31920 | Two-component response regulator ARR10 | OS02G0139400 | Squamosa promoter-binding-like protein 3 |
| 70 | AT2G01760 | Two-component response regulator ARR14 | OS02G0174100 | Squamosa promoter-binding-like protein 4 |
| 71 | AT5G58080 | Two-component response regulator ARR18 | OS02G0177300 | Squamosa promoter-binding-like protein 5 |
| 72 | AT4G16110 | Two-component response regulator ARR2 | OS03G0833300 | Squamosa promoter-binding-like protein 6 |
| 73 | AT4G18020 | Two-component response regulator-like APRR2 | OS04G0551500 | Squamosa promoter-binding-like protein 7 |
| 74 | AT1G55600 | WRKY transcription factor 10 | OS05G0408200 | Squamosa promoter-binding-like protein 9 |
| 75 | AT4G31550 | WRKY transcription factor 11 | OS06G0275600 | TA1 protein |
| 76 | AT2G44745 | WRKY transcription factor 12 | OS08G0524800 | TA1 protein |
| 77 | AT4G39410 | WRKY transcription factor 13 | OS08G0536800 | TA1 protein |
| 78 | AT1G30650 | WRKY transcription factor 14 | OS09G0474100 | TA1 protein |
| 79 | AT2G23320 | WRKY transcription factor 15 | OS12G0580300 | TATA-binding protein TBP2 |
| 80 | AT5G45050 | WRKY transcription factor 16 | OS10G0432300 | TATA-box binding protein 1 |
| 81 | AT2G24570 | WRKY transcription factor 17 | OS01G0812000 | Transcription factor GAMYB |
| 82 | AT4G31800 | WRKY transcription factor 18 | OS04G0194600 | Transcription factor PCF1 |
| 83 | AT4G12020 | WRKY transcription factor 19 | OS08G0544800 | Transcription factor PCF2 |
| 84 | AT5G56270 | WRKY transcription factor 2 | OS11G0175700 | Transcription factor PCF3 |
| 85 | AT4G26640 | WRKY transcription factor 20 | OS02G0706600 | zinc finger homeodomain protein 1 |
| 86 | AT2G30590 | WRKY transcription factor 21 |  |  |
| 87 | AT4G01250 | WRKY transcription factor 22 |  |  |
| 88 | AT2G47260 | WRKY transcription factor 23 |  |  |
| 89 | AT2G30250 | WRKY transcription factor 25 |  |  |
| 90 | AT5G07100 | WRKY transcription factor 26 |  |  |
| 91 | AT5G52830 | WRKY transcription factor 27 |  |  |
| 92 | AT4G18170 | WRKY transcription factor 28 |  |  |
| 93 | AT2G03340 | WRKY transcription factor 3 |  |  |
| 94 | AT5G24110 | WRKY transcription factor 30 |  |  |
| 95 | AT4G22070 | WRKY transcription factor 31 |  |  |
| 96 | AT4G30935 | WRKY transcription factor 32 |  |  |
| 97 | AT2G38470 | WRKY transcription factor 33 |  |  |
| 98 | AT4G26440 | WRKY transcription factor 34 |  |  |
| 99 | AT2G34830 | WRKY transcription factor 35 |  |  |
| 100 | AT1G69810 | WRKY transcription factor 36 |  |  |
| 101 | AT5G22570 | WRKY transcription factor 38 |  |  |
| 102 | AT3G04670 | WRKY transcription factor 39 |  |  |
| 103 | AT1G13960 | WRKY transcription factor 4 |  |  |
| 104 | AT1G80840 | WRKY transcription factor 40 |  |  |
| 105 | AT4G04450 | WRKY transcription factor 42 |  |  |
| 106 | AT2G46130 | WRKY transcription factor 43 |  |  |
| 107 | AT2G37260 | WRKY transcription factor 44 |  |  |
| 108 | AT3G01970 | WRKY transcription factor 45 |  |  |
| 109 | AT2G46400 | WRKY transcription factor 46 |  |  |
| 110 | AT4G01720 | WRKY transcription factor 47 |  |  |
| 111 | AT5G49520 | WRKY transcription factor 48 |  |  |
| 112 | AT5G45260 | WRKY transcription factor 52 |  |  |
| 113 | AT4G23810 | WRKY transcription factor 53 |  |  |
| 114 | AT2G40750 | WRKY transcription factor 54 |  |  |
| 115 | AT2G40740 | WRKY transcription factor 55 |  |  |
| 116 | AT1G64000 | WRKY transcription factor 56 |  |  |
| 117 | AT1G69310 | WRKY transcription factor 57 |  |  |
| 118 | AT3G01080 | WRKY transcription factor 58 |  |  |
| 119 | AT1G62300 | WRKY transcription factor 6 |  |  |
| 120 | AT2G25000 | WRKY transcription factor 60 |  |  |
| 121 | AT1G18860 | WRKY transcription factor 61 |  |  |
| 122 | AT1G29280 | WRKY transcription factor 65 |  |  |
| 123 | AT1G80590 | WRKY transcription factor 66 |  |  |
| 124 | AT1G66550 | WRKY transcription factor 67 |  |  |
| 125 | AT3G58710 | WRKY transcription factor 69 |  |  |
| 126 | AT4G24240 | WRKY transcription factor 7 |  |  |
| 127 | AT3G56400 | WRKY transcription factor 70 |  |  |
| 128 | AT1G29860 | WRKY transcription factor 71 |  |  |
| 129 | AT5G15130 | WRKY transcription factor 72 |  |  |
| 130 | AT5G28650 | WRKY transcription factor 74 |  |  |
| 131 | AT5G13080 | WRKY transcription factor 75 |  |  |
| 132 | AT5G46350 | WRKY transcription factor 8 |  |  |
| 133 | AT1G68150 | WRKY transcription factor 9 |  |  |
| 134 | AT1G27730 | ZAT10, salt tolerance zinc finger |  |  |
| 135 | AT3G19580 | zinc-finger protein 2 |  |  |
| 136 | AT5G43170 | zinc-finger protein 3 |  |  |
