# Supplementary material for: Multiple Herbicide Resistance in Lolium multiflorum and Identification of Conserved Regulatory Elements of Herbicide Resistance Genes
Source: Front Plant Sci. 2016 Aug 5;7:1160. doi: 10.3389/fpls.2016.01160 (PMC4974277; doi:10.3389/fpls.2016.01160)
Supplement: Supplementary file 4 [file Image1.PDF]

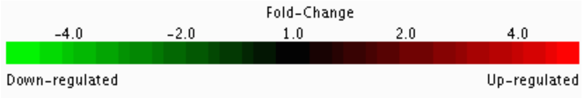

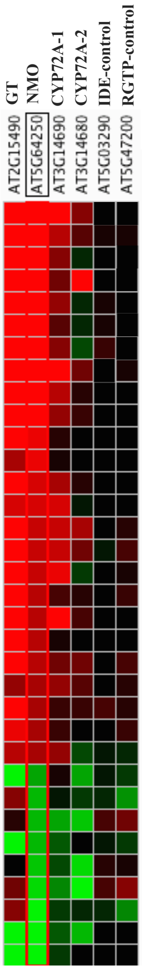

	Log(2)-ratio	| 3 | Fold-Change	0.001 p-value
fenclorim (4h) / solvent treated root culture samples (4h)	4.02	16.13	<0.001
phytoprostane A1 (Col-0) / solvent treated (Col-0) seedlings	3.16	9.11	<0.001
arsenate study 3 (30μM As(V)) / shift 5μM Pi to 30μM Pi	3.16	9.03	<0.001
salicylic acid study 3 / mock treated seedlings	3.06	8.45	<0.001
TIBA / mock treated seedlings	3.08	8.44	<0.001
arsenate study 3 (30μM As(V)) / P deficiency study 6 (5μM Pi)	2.82	7.09	<0.001
CMP (4h) / solvent treated root culture samples (4h)	2.81	7.00	<0.001
fenclorim (24h) / solvent treated root culture samples (24h)	2.72	6.73	<0.001
4-thiazolidinone/acetic acid / solvent treated seedlings	2.63	6.20	<0.001
furyl acrylate ester / solvent treated seedlings	2.44	5.43	<0.001
shift 28°C to 19°C study 2 (35S:RPS4-HS) / 28°C (35S:RPS4-HS)	2.40	5.27	<0.001
shift 28°C to 19°C study 2 (35S:RPS4-HS rrs1-11) / 28°C (35S:RPS4-HS rrs1-...	2.34	5.09	<0.001
CMP (24h) / solvent treated root culture samples (24h)	2.12	4.36	<0.001
arsenate (Ws-2) / untreated Ws-2 root samples	2.10	4.29	<0.001
dicamba (10h) / H2O treated seedling samples (10h)	1.97	3.91	<0.001
drought study 15 (camta1-3) / untreated camta1-3 root samples	1.95	3.87	<0.001
oligomycin (4h) / solvent treated seedling culture samples (4h)	1.94	3.81	<0.001
low light + DBMIB (0.5h) / low light study 4 (6h)	1.88	3.72	<0.001
MeJa study 3 (2h) / solvent treated cell culture samples (2h)	1.83	3.55	<0.001
ozone / air treated seedlings	1.81	3.53	<0.001
low light + DBMIB (2h) / low light study 4 (6h)	1.77	3.41	<0.001
camta1-3 / Col-0	1.74	3.35	<0.001
Pep2 study 2 (bak1-3) / mock treated seedling samples (bak1-3)	1.72	3.28	<0.001
ABA study 13 (Col) / mock treated Col whole plant samples	1.71	3.28	<0.001
hypoxia study 2 (late) / untreated seedlings (late)	1.63	3.11	<0.001
C24 / Col	-1.60	-3.04	<0.001
cycloheximide study 4 (pBeaconRFP_GR-ABI3) / mock treated root protoplast s...	-1.83	-3.58	<0.001
germination (48h) / seed desiccation	-1.85	-3.61	<0.001
P. syringae pv. tomato study 2 (DC3000 hrcC-) / P. syringae pv. tomato study ...	-1.85	-3.62	<0.001
germination (12h) / seed desiccation	-1.93	-3.80	<0.001
germination (24h) / seed desiccation	-2.22	-4.66	<0.001
cycloheximide / dexamethasone study 2 (pBeaconRFP_GR-ABI3) / mock treate...	-2.30	-4.94	<0.001
BL study 2 (brx) / mock treated seedlings (brx)	-2.49	-5.64	<0.001
35S:RPS4-HS eds1-2 / 35S:RPS4-HS	-2.64	-6.25	<0.001

Fig S1: Gene expression pattern of *Lolium multiflorum* orthologs of GT, NMO, CYPs along with control genes in *Arabidopsis thaliana*. Heat map of expression of selected genes in response to various external conditions such as chemicals were analyzed using Genevestigator perturbation tool. Relative expression of the genes was represented in $\log_2$ ratio and significant change in expression were filtered out based on NMO, *p*-value <0.001 and fold change greater than 3. Expression of HMR genes strongly induced in response to various chemicals including herbicides and herbicide safeners.
