# Supplementary material for: Multiple Herbicide Resistance in Lolium multiflorum and Identification of Conserved Regulatory Elements of Herbicide Resistance Genes
Source: Front Plant Sci. 2016 Aug 5;7:1160. doi: 10.3389/fpls.2016.01160 (PMC4974277; doi:10.3389/fpls.2016.01160)
Supplement: Supplementary file 5 [file Image2.PDF]

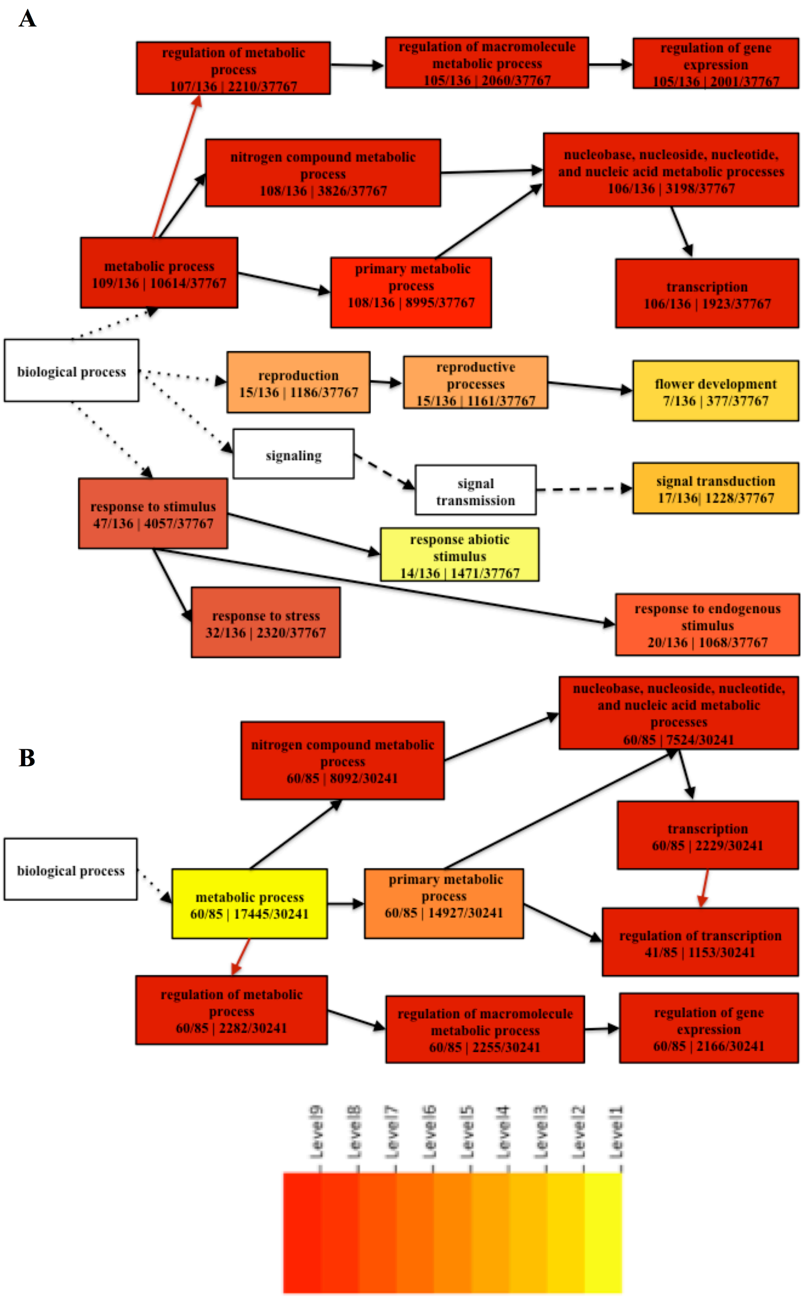

Fig S2: Biological processes of TFs associated to the promoters of all four herbicide metabolism genes. The hierarchical graphs of over-represented terms of *A.thaliana* (A) and *O.sativa* (B) by Singular Enrichment Analysis (SEA) using AgriGO (http://bioinfo.cau.edu.cn/agriGO/index.php) were drawn manually. Boxes in the graph represent biological processes labeled by their definition and statistical information. The significant term ($P < 0.05$) are marked with color while non-significant terms are shown as white boxes. The degree of color saturation of a box is positively correlated to the enrichment level of the term. Solid, dashed and dotted lines represent two, one and zero enrichment terms at both ends connected by line, respectively while red color line represents positive correlation to the enrichment level of term. The rank direction of graph runs from left to right.
