# Supplementary material for: Multiple Herbicide Resistance in Lolium multiflorum and Identification of Conserved Regulatory Elements of Herbicide Resistance Genes
Source: Front Plant Sci. 2016 Aug 5;7:1160. doi: 10.3389/fpls.2016.01160 (PMC4974277; doi:10.3389/fpls.2016.01160)
Supplement: Supplementary file 6 [file Image3.PDF]

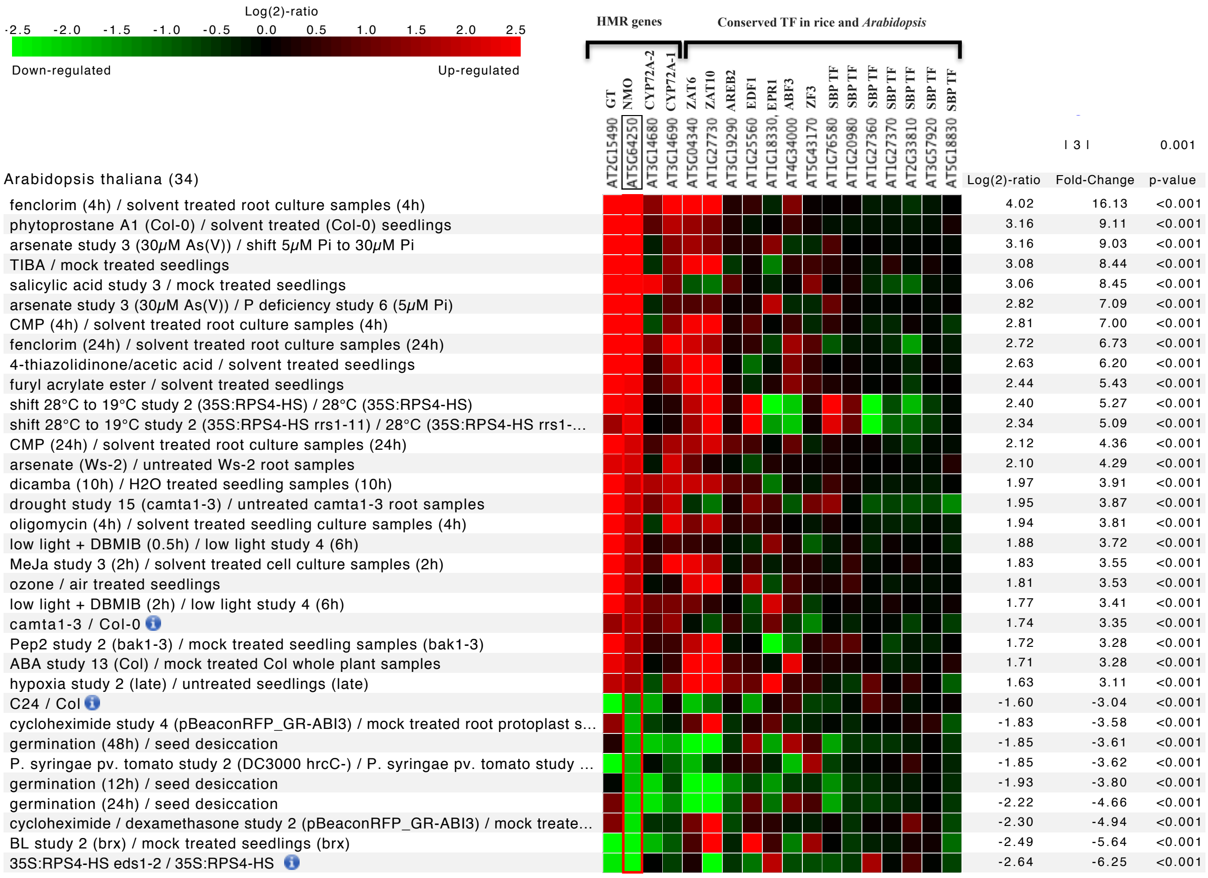

Fig S3: Expression pattern of HMR genes along with identified conserved transcription factors. Heat map of the expression of identified conserved TFs along with HMR genes under various chemical stress were analyzed using Genevestigator perturbation tool. Relative expression of the genes was represented in $\log_2$ ratio and significant change in expression were filtered out based on NMO, *p*-value <0.001 and fold change greater than 3. Expression of these genes strongly induced in response to various chemicals including herbicides and herbicide safeners
